# Application of Laser-Induced Bone Therapy by Carbon Dioxide Laser Irradiation in Implant Therapy

**DOI:** 10.1155/2012/409496

**Published:** 2012-02-06

**Authors:** Takahiro Naka, Satoshi Yokose

**Affiliations:** Division of Operative Dentistry, Department of Conservative Dentistry, Ohu University School of Dentistry, Ohu University School of Dentistry, 31-1 Misumido, Tomita machi, Koriyama, Fukushima 963-8611, Japan

## Abstract

This study evaluated the application of laser-induced bone therapy (LIBT) to reduce implant healing time in rat tibia. Twenty 10-week-old female Sprague-Dawlay rats were used. The rats received laser irradiation (laser group) or sham operation (control group) on either side of the tibia. Five days after invasion, titanium implants were inserted in proximal tibia. Five, 10, and 20 days after implant placement, tibiae were collected. After taking micro-CT and performing a torque test, the tibiae were decalcified and 8-**μ**m-thick sections were prepared. Specimens were stained with hematoxylin and eosin. *Results*. Micro-CT images, removal torque values, and histomorphometric analysis data demonstrated a significantly accelerated bone formation in the laser group earlier in the healing process. *Conclusion*. The use of laser irradiation was effective in promoting bone formation and acquiring osseointegration of titanium implants inserted in rat tibia. LIBT may be suitable for use in implant therapy.

## 1. Introduction

Bone formation in peri-implant tissue is a key factor in acquiring osseointegration and maintaining implant stability. However, a longer period for healing and acquiring osseointegration is needed in order to add occlusal load. It has been suggested that a standard healing period of at least 3 months in the mandible and 6 months in the maxilla is needed before implant loading [1–3]. Thus, to speed up the rehabilitation process is still a challenging and important clinical aim.

The skeleton that adapts to mechanical usage and mechanical loading promotes bone formation and remodeling, which is commonly referred to as “Wolff's Law” [4, 5] and Frost's theory [6–8]. Mechanical strains such as pulsed electromagnetic fields [9, 10] and low-intensity pulsed ultrasound [11, 12] are widely accepted. Currently, lasers are commonly used in medical and dental treatment. Clinical applications of lasers are largely divided into low reactive level laser therapy (LLLT) and high reactive level laser therapy (HLLT). LLLT provides photobiological and photochemical effects. LLLT enhanced healing, especially in soft tissues [13, 14] such as healing of ulcers [15] and other wounds [16]. Recent research demonstrated that the enhancement of functional attachment of bone-to-titanium implants and promotion of bone mineralization could be achieved by LLLT [17–21]. This would allow the implant to be loaded after a shorter period, reducing the treatment time. A positive effect of LLLT on osseointegration of implants and maturation of peri-implant bone was mainly obtained with Gallium Aluminum Arsenide (GaAlAs) laser [17, 18, 20]. HLLT is useful for cutting biologic materials and producing coagulation necrosis in target tissues with a subsequent reaction in the surrounding tissue. When HLLT is applied to hard tissues such as tooth or bone, carbon dioxide laser (CO_2_ laser) induces extreme cracking and charring of surrounding enamel, dentin, and bone. After HLLT CO_2_ laser irradiation on rat tibia, disappearance and shrinkage of osteocytes within the lacunae have been observed [22–27]. New bone formation was also observed at the tibial wall adjacent to the marrow cavity under the laser-irradiated cortex [27, 28]. However, HLLT thermal damage results in extensive cell mediated resorption of bone or sequestration of dead bone [29], thus severely limiting the use of HLLT on bone. Currently, CO_2_ laser is common laser used in dental clinics and it also enables rapid and precise tissue destruction, reduces bleeding and postoperative pain, and results in low morbidity, minimal scarring, and wound contracture [30]. However, the application of CO_2_ laser in dental treatment is restricted to the treatment of soft tissue.

This time, we hypothesized that bone formation occurring within the marrow mediated by HLLT CO_2_ laser irradiation would accelerate the osseointegration process and reduce healing time. To evaluate our hypothesis, an animal study using functional and morphological analysis was performed.

## 2. Material and Methods

### 2.1. Experimental Design

**Figure 1:**
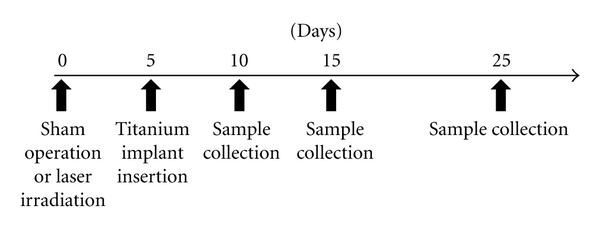
Experimental protocol. Time schedule of bur/laser injury, implant placement, and subsequent healing periods.

The care and use of animals followed “The Guidelines for the Care and Use of Animals” approved by Ohu University in accordance with the principles of the NIH guidelines (approval date: 5/14/2009; Approval number: 2). Twenty 10-week-old female Sprague-Dawley rats were purchased from Crea Japan (Tokyo, Japan) and used as the experimental model in this study. The right tibia of all rats was treated with laser irradiation, and left tibia was sham operated. Five days after laser irradiation and sham operation, titanium implants were placed. Briefly, under general anesthesia, the surface of the proximal metaphases of the tibiae was exposed by an incision approximately 10 mm in length. Under constant saline irrigation, a bicortical implant bed was drilled with a dental bur at a rotary speed not exceeding 1500 rpm, and the implant was placed until the screw thread completely penetrated the bone cortex. After installation, the soft tissue was replaced and sutured. After these procedures, the animals were housed with free access to water and provided a diet. Care was taken to avoid unnecessary stress and discomfort to the animal throughout the experimental period. Five animals were sacrificed 5, 10, or 20 days after implantation, and tibiae containing implants were collected (Figure 1).

### 2.2. CO_2_ Laser

In this experiment, CO_2_ laser (NANOLASER GL-III, GC Co., Tokyo, Japan, and OPELASER Lite, Yoshida Co., Tokyo, Japan) was used at a wavelength of 10.6 *μ*m, a diameter of 1.70 mm, output of 1.0 W, and a continuous wave form. The laser beam was focused by maintaining 10 mm delivery tip-to-target surface distance. Total irradiated energy was 220.4 J/cm^2^.

### 2.3. Titanium Implant

Screw-shaped implants made from commercially pure titanium were used in this study (Nishimura Co., Ltd., Fukui, Japan). The total length of each implant was 2 mm, thread diameter 1.4 mm, and pitch 0.6 mm. Implants were cleaned in absolute ethanol in an ultrasonic bath and sterilized by autoclaving.

### 2.4. Microtomographic Histomorphometry (Micro-CT)

After sacrificing the rats, tibiae with titanium implants were collected, and microtomographic histomorphometry was performed with a high-resolution micro-CT system (TOSCANER-30000, Toshiba IT and Control Systems Co., Tokyo, Japan). The Computed tomography parameters were as follows: (1) the image pixel size was set to 1024 × 1024; (2) the slice thickness was set to 0.05 mm; (3) the image magnification was set to 10x; (4) the X-ray tube voltage was set to 100 kV; (5) the anode electrical current was set to 80 *μ*A. Three dimensional images were reconstructed using the microreconstruct software (Simplant Pro, Materialise Dental Japan Inc., Tokyo, Japan).

### 2.5. Torque Test

After stabilization of the implanted tibia, the force needed to unscrew the implants was measured using a Tohnichi Torque driver FTD2-S (Tohnichi Mfg. Co., Ltd., Tokyo, Japan). It has a round dial gauge with a pointer to read the peak value. Peak value when the rupture occurred between implant and bone was recorded, and the mean torque measurements were calculated for each implant inserted into the tibia specimen.

### 2.6. Histomorphometric Procedure

After removing the titanium implants, collected tibiae were fixed in 10% phosphate-buffered neutral formalin (pH 7.4) (Wako Pure Chemical Industries, Ltd., Osaka, Japan), decalcified in 0.5 mol/L EDTA (pH 7.5) (Wako Pure Chemical Industries) for 2 weeks at 4°C, dehydrated in an ethanol (Wako Pure Chemical Industries) series, washed in xylene (Wako Pure Chemical Industries), and then embedded in paraffin. Decalcified 8-*μ*m thick sections were made and stained with hematoxylin and eosin (H&E) for general morphological analysis.

### 2.7. Statistical Analysis

For biomechanical testing analysis, a two-way analysis of variance was used to examine the influence on osseointegration of (1) laser or sham operated and (2) the length of the healing period. Differences with a *P* value less than 0.05 were considered significant.

## 3. Results

### 3.1. Osteoid Formation (Figure 2)

**Figure 2:**
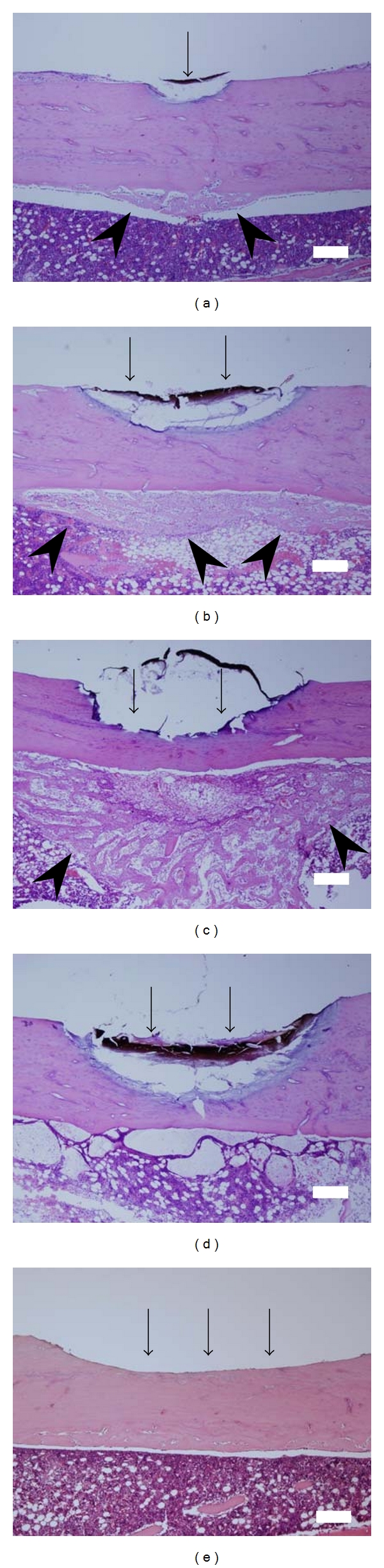
Comparison of osteoid formation in the bone marrow space in the laser-irradiated group and bur-injured group 5 days after treatment. (a) laser-irradiated tibia. Energy density = 88.2 J/cm^2^, (output: 1.0 W, irradiation time: 2 sec). (b) laser-irradiated tibia. Energy densities = 220.5 J/cm^2^, (output: 1.0 W, irradiation time: 5 sec). (c) laser-irradiated tibia. Energy densities = 441.0 J/cm^2^, (output: 1.0 W, irradiation time: 10 sec). (d) laser-irradiated tibia. Energy densities = 661.5 J/cm^2^, (output: 1.0 W, irradiation time: 15 sec). (e) bur-injured tibia. (a–d) laser-irradiated tibia showed an ablation defect, carbon deposits, and numerous empty osteocytic lacunae. Moreover, newly formed trabecular bone was observed on the marrow side of the laser-treated site (a–c). (e) bur-injured tibia showed a slight amount of reactive bone formation on the endosteal surface. H&E stain with 40x magnification. Bar = 200 *μ*m. Arrow indicates laser-irradiated or bur-injured site. Arrow heads indicate osteoid formation site.

To examine the correlation between total irradiated energy and osteoid formation capacity, we evaluated four kinds of energy densities, 88.2, 220.4, 441.0, and 661.5 J/cm^2^. Five days after laser irradiation, a char layer, empty osteocytic lacunas were observed. The osteocytic lacunae in most of the cortical bone appeared to be devoid of osteocytes because the typical blue-purple staining of osteocytic nuclei was absent on H&E staining. Moreover, as energy densities increased, the depth of ablation and width of surface damage increased (Figures 2(a)–2(d)). Osteoid formation just under the irradiated cortical bone was observed in the 88.2, 220.5, and 441.0 J/cm^2^ groups tibia (Figures 2(a)–2(c)). In the 441.0 J/cm^2^ group tibia, no osteoid formation was observed (Figure 2(d)). In the bur injured tibia, a cortical bone defect and a small amount of reactive bone formation in the bone marrow space were observed (Figure 2(e)). From this observation, we used 220.5 J/cm^2^ to this experiment.

### 3.2. Micro-CT Observation of Tibia (Figure 3)

Ten days after injury (5 days after implantation), little radiopacity was observed around the implant body in the bur-implant group tibia. In the laser group tibia, evident radiopacity was observed around the titanium implant.

Fifteen days after injury (10 days after implantation), beginning of evident radiopacity was observed around titanium implant body in the control group. In the laser group tibia, increased radiopacity was observed around the titanium implant.

Twenty-five days after injury (20 days after implantation), obvious radiopacity, though thinner than that in the laser group, was observed around the implant body. In the laser group tibia, thick radiopacity was observed around the implant body.

### 3.3. Removal Torque Test (Figure 4)

Functional attachment of the integration between implants and bone was evaluated using a torque test with torque drivers.

Ten days after injury (5 days after implant placement), the average removal torque was 0.63 ± 0.18 N cm for the control group and 1.36 ± 0.15 N cm for the laser group. There was a significant difference between the laser and control groups.

Fifteen days after injury (10 days after implant placement), the average removal torque was 0.68 ± 0.15 N cm for the control group and 1.65 ± 0.21 N cm for the laser group. There was a statistically significant difference between the laser and control groups.

Twenty-five days after injury (20 days after implant placement), the average removal torque was 0.89 ± 0.16 N cm for the control group and 1.87 ± 0.28 N cm for the laser group. There was a significant difference between the laser and control groups. Moreover, there were significant differences between 25-day value of control group and 10- or 15-day value of laser group.

### 3.4. Histological Findings (Figure 5)

#### 3.4.1. Day 10 (Figures 5(a) and 5(d))

**Figure 5:**
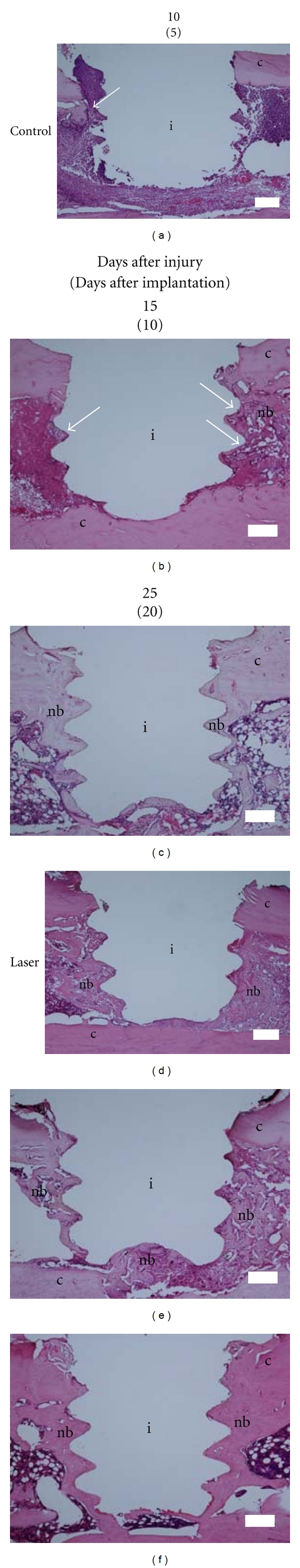
H&E staining observation. (a, d): 5 days after implantation (10 days after sham operation or laser irradiation). (b, e): 10 days after implantation (15 days after sham operation or laser irradiation). (c, f): 20 days after implantation (25 days after sham operation or laser irradiation). (a–c): control group tibia. (d–f): laser group tibia. In the early healing period (10 days after injury), in the control group, there was limited bone formation around the implant body, while in the laser group, there was obvious bone formation along the inserted implant. Fifteen days after injury, in the control group, tibia showed little osteoid formation around the implant body. In the laser group, thick and abundant bone formation was evident along the inserted implant body. Twenty-five days after injury, in the control group, tibia showed newly formed cortical bone, but the trabecular bone was thinner than that in the laser group tibia. In the laser group, thick cortical bone was connected to the previously existing cortex. H&E stain with 40x magnification. c: cortical bone, i: implant cavity, nb: newly formed bone, bar = 200 *μ*m.

Ten days after injury (5 days after titanium implant insertion), hematoma, soft tissue, and little bone fragment formation were observed around the inserted titanium implant in the control group tibia (Figure 5(a)). In the laser group tibia, a large amount of bone matrix had formed along the bone-implant interface (Figure 5(d)).

#### 3.4.2. Day 15 (Figures 5(b) and 5(e))

Fifteen days after injury (10 days after titanium implant insertion), formation of woven bone was observed around the inserted titanium implant in the control group tibia (Figure 5(b)). In the laser group, most of the implant surface was in direct contact with the new woven bone (Figure 5(e)).

#### 3.4.3. Day 25 (Figures 5(c) and 5(f))

Twenty-five days after injury (20 days after titanium implant insertion), the implant surface was also covered with newly formed bone in the control group tibia. But the trabecular bone was thinner than that in the laser group (Figure 5(c)). In the laser group, most of the implant surface was covered with thick newly formed bone lamella, which was connected with preexisting bone by newly formed trabeculae. Moreover, osteocytes were apparent in the newly formed bone matrix surrounding the implant (Figure 5(f)).

## 4. Discussion

CO_2_ laser emits a beam of energy that lases tissues in a noncontact mode. HLLT CO_2_ laser irradiation is known to produce a photobiodestructive reaction inducing cellular vaporization, whereas LLLT CO_2_ laser therapy generates a photobioactive reaction (PAR) stimulating cellular proliferation and differentiation [31]. CO_2_ laser has a wavelength of 10.6 *μ*m, which falls within the specific absorption spectrum for calcium hydroxyapatite, 9.0 to 11.0 *μ*m [32]. Moreover, previous report indicated that when laser irradiation was performed on the cortical bone, only CO_2_ laser could induce newly bone formation in the marrow cavity [26]. Based on this observation, the mineral components of bone are expected to exhibit maximal absorption of the laser energy. It seems reasonable to assume that CO_2_ laser irradiation of the bone tissue would be suitable for bone regeneration therapy. However, it is commonly accepted that CO_2_ laser is unable to use hard tissue treatment.

The bone tissue that received laser irradiation demonstrated acceleration of bone formation. Bone formation induced by CO_2_ laser has also been reported previously [24–26, 33, 34]. As cellular mechanisms of this reactive bone formation have been obscure, we speculated that osteocytes have been capable of playing a part. In fact, Tasumi et al. reported that bone formation in tibiae of transgenic mouse model in which specific ablation of osteocytes have been accelerated drastically [35]. They pointed out that mature osteocytes express *Sclerostin*, the negative regulator of osteoblastic bone formation by antagonizing BMP and Wnt signal. Moreover, the expression of *Sclerostin* was decreased followed by osteocytes ablation in the transgenic mouse model, which may cause stimulation of bone formation. In this present study, judging from osteocytic appearances with pyknotic, shrunken, and displaced cells within their lacunae, laser irradiation locally damaged osteocytes. Damaged osteocytes mediated by laser irradiation also decreased *sclerostin* expression; the bone formation could be stimulated. However, it is necessary to analyze what is the negative and/or positive regulator of osteocyte-derived factors induced by laser irradiation. Further investigation will be required to clarify this point.

However, laser irradiation also induced not only damage of cortex but also induced the inflammatory reaction and degeneration of bone marrow. In this study, we inserted titanium implant to the damaged cortex and bone marrow. To achieve the early healing and lording, osseointegration of screw neck to cortical bone is important. Additional studies are needed to clarify the effective irradiation energy to minimize the heat damaged cortical bone and bone marrow inflammatory reaction in order to apply this method to dental implant therapy.

To confirm whether laser-induced bone therapy (LIBT) was effective in implant therapy, we inserted a titanium implant into woven bone and evaluated the bone formation. Although an implant has the ability to induce bone formation around itself, we hypothesized that bone formation occurring within the marrow prior to implant insertion would accelerate the osseointegration process and reduce healing time. To evaluate our hypothesis, we used histomorphological and clinical parameters to evaluate the degree of bone formation and osseointegration. Micro-CT image analysis [36], removal torque measurements [37–43], and histomorphometric evaluation [21, 43–47] are currently considered standard analyses in implant research. In the early healing stage, advancement of histomorphological changes and significantly increased functional analysis data were observed in the laser-implant group. These results may be attributed to the laser-mediated bone formation in the marrow area. These results suggest that preoperative HLLT treatment may promote formation of bone tissue with a tighter mesh of trabeculae, which promotes early osseointegration.

Many signaling molecules, such as growth factors and hormones, are involved in bone metabolism [48, 49], especially around the titanium implant [50]. Further investigations are needed to focus on the underlying biological mechanisms, which induce osseointegration in implant therapy when using LIBT.

**Figure 6:**
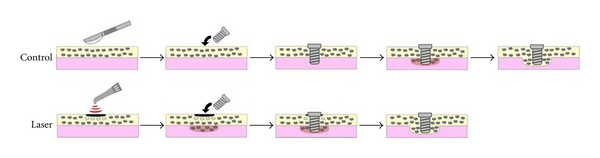
Summary of the healing processes in the control group and laser group. Laser irradiation-induced bone formation in the bone marrow and laser group showed earlier osseointegration. The control group also acquired osseointegration, but the healing period was longer than that of the laser group.

In this experimental model, the application of LIBT before implant insertion may promote bone formation and facilitate osseointegration of titanium implants (Figure 6). The introduction of LIBT in implant treatment seems feasible and may be of therapeutic benefit in accelerating healing.

## Figures and Tables

**Figure 3:**
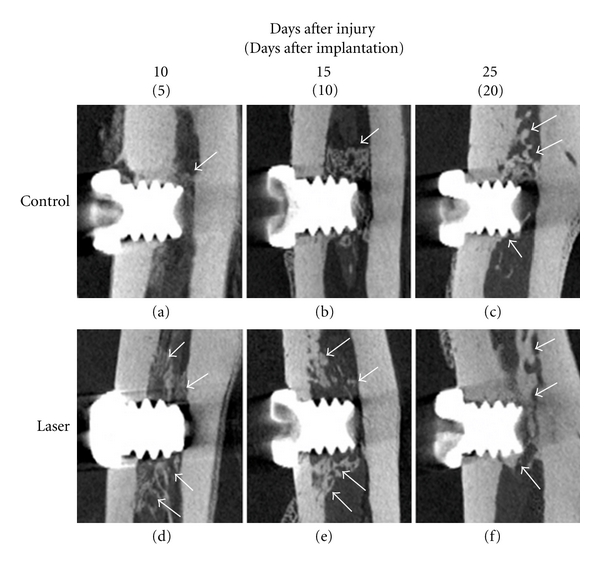
Micro-CT images. (a, d): 5 days after implantation (10 days after sham operation or laser irradiation). (b, e): 10 days after implantation (15 days after sham operation or laser irradiation). (c, f): 20 days after implantation (25 days after sham operation or laser irradiation). (a–c): control group tibia. (d–f): laser group tibia. Arrow indicates newly formed bone.

**Figure 4:**
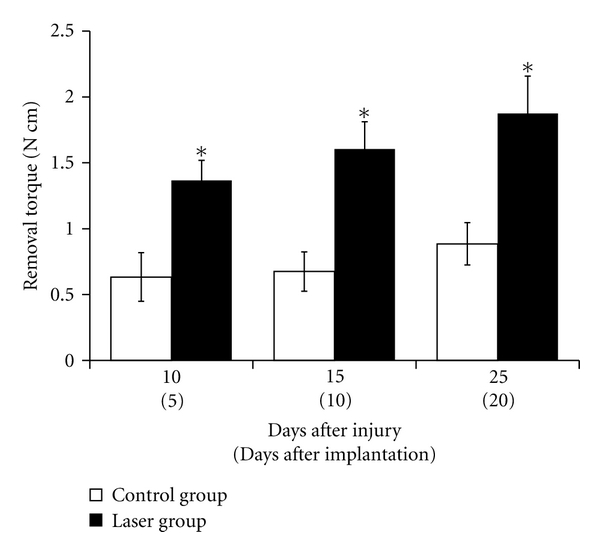
Removal torque examination of titanium implant. Removal torque values were measured at different time points. Mean ± SE (N cm) of torque forces in laser group and control group. Five days after implantation, significant difference was observed between the control and laser groups. Until twenty days after implantation, there was still a significant difference between the two groups. *P < 0.05.
